# Monoallelic *POLR3A* Variants Cause Early‐Onset Peripheral Neuropathy

**DOI:** 10.1002/ana.78268

**Published:** 2026-06-08

**Authors:** Luiza L. P. Ramos, Jevin M. Parmar, Robin Wijngaard, Bianca R. Grosz, Tamas Lazar, Ligia Mateiu, Steve Vucic, Kishore R. Kumar, Dennis Yeow, Laura I. Rudaks, Lonneke de Boer, Annemarie de Vreugd, David A. Koolen, Thatjana Gardeitchik, Anita Cairns, Krishnan Iyengar, Fernando Kok, Fernanda Barbosa Figueiredo, Alzira Alves de Siqueira Carvalho, Luiz S. Mageste Barbosa, Rodrigo Rezende Arantes, Tyler Rehbein, Jordan E. Bontrager, Elizabeth P. Wood, Janet E. Sowden, Gavin Monahan, Meutia Kumaheri, Ivy Cuijt, Melina Ellis, Gonzalo Perez‐Siles, Elyshia McNamara, Ronald van Beek, Celine B. Meijers, Ivaylo Tournev, Stephan Zuchner, Shoshana J. Wodak, Clara D. M. van Karnebeek, Nigel Laing, Liana N. Semcesen, David A. Stroud, David N. Herrmann, Velina Guergueltcheva, Marina L. Kennerson, Machteld M. Oud, Gianina Ravenscroft, Ayse Candayan, Albena Jordanova

**Affiliations:** ^1^ Molecular Neurogenomics Group, VIB Center for Molecular Neurology Antwerp Belgium; ^2^ Department of Biomedical Sciences University of Antwerp Antwerp Belgium; ^3^ Harry Perkins Institute of Medical Research, Centre for Medical Research, University of Western Australia Nedlands Western Australia Australia; ^4^ Department of Human Genetics Radboud University Medical Center Nijmegen The Netherlands; ^5^ Donders Institute for Brain, Cognition and Behaviour, Radboud University Medical Center Nijmegen The Netherlands; ^6^ United for Metabolic Diseases (UMD) Amsterdam The Netherlands; ^7^ Northcott Neuroscience Laboratory, ANZAC Research Institute Sydney New South Wales Australia; ^8^ School of Medical Sciences, Faculty of Medicine and Health, The University of Sydney Camperdown New South Wales Australia; ^9^ VIB‐VUB Center for Structural Biology, VIB Brussels Belgium; ^10^ Structural Biology Brussels (SBB), Department of Bioengineering Vrije Universiteit Brussel (VUB) Brussels Belgium; ^11^ Department of Medical Genetics University of Antwerp Antwerp Belgium; ^12^ Brain and Nerve Research Center, Concord Clinical School, University of Sydney Sydney New South Wales Australia; ^13^ Neurology Department Concord Repatriation General Hospital Sydney New South Wales Australia; ^14^ Neurology Department and Molecular Medicine Laboratory Concord Repatriation General Hospital, Sydney Local Health District and NSW Health Pathology Sydney New South Wales Australia; ^15^ Translational Neurogenomics Group, Genomic and Inherited Disease Program, The Garvan Institute of Medical Research Darlinghurst New South Wales Australia; ^16^ School of Clinical Medicine, UNSW Medicine & Health, St. Vincent's Healthcare Clinical Campus, Faculty of Medicine and Health, UNSW Sydney Sydney New South Wales Australia; ^17^ Sydney Medical School, University of Sydney Camperdown New South Wales Australia; ^18^ Clinical Genetics Unit, Royal North Shore Hospital Sydney New South Wales Australia; ^19^ Department of Pediatrics Amalia Children's Hospital, Radboud University Medical Center Nijmegen The Netherlands; ^20^ Neurosciences Department Queensland Children's Hospital Brisbane Queensland Australia; ^21^ Department of Anatomical Pathology Royal Brisbane and Women's Hospital Brisbane Queensland Australia; ^22^ Department of Neurology University of Sao Paulo School of Medicine Sao Paulo Brazil; ^23^ Mendelics Genomic Analysis Sao Paulo Brazil; ^24^ Neuromuscular Reference Center, Citadella Hospital, University of Liege Liege Belgium; ^25^ Neuromuscular Disease Centre, Department of Neurosciences University Center ABC Santo André Brazil; ^26^ Neuromuscular Disease Centre, Department of Neurology University Hospital of the Federal University of Minas Gerais Belo Horizonte Brazil; ^27^ Medical Genetics Service, University Hospital of the Federal University of Minas Gerais Belo Horizonte Brazil; ^28^ Department of Neurology University of Rochester Rochester NY; ^29^ Genomics Pillar, Garvan Institute of Medical Research Sydney New South Wales Australia; ^30^ Centre for Population Genomics, Garvan Institute of Medical Research Sydney New South Wales Australia; ^31^ Murdoch Children's Research Institute Melbourne Victoria Australia; ^32^ St. Vincent Clinical School, University of New South Wales Sydney New South Wales Australia; ^33^ Department of Neurology Clinic of Nervous Diseases, UMBAL Aleksandrovska, Medical University‐Sofia Sofia Bulgaria; ^34^ Department of Cognitive Science and Psychology New Bulgarian University Sofia Bulgaria; ^35^ Dr. John T. Macdonald Foundation Department of Human Genetics University of Miami Miller School of Medicine Miami FL; ^36^ John P. Hussman Institute for Human Genomics, University of Miami Miller School of Medicine Miami FL; ^37^ Department of Pediatrics and Human Genetics Emma Center for Personalized Medicine, Amsterdam University Medical Center Amsterdam The Netherlands; ^38^ Department of Biochemistry and Pharmacology Bio21 Molecular Science and Biotechnology Institute, University of Melbourne Parkville Victoria Australia; ^39^ Victorian Clinical Genetics Services Melbourne Victoria Australia; ^40^ Clinic of Neurology, University Hospital Sofiamed Sofia Bulgaria; ^41^ Department of Neurology Sofia University “St. Kliment Ohridski” Sofia Bulgaria; ^42^ Molecular Medicine Laboratory, Concord Repatriation General Hospital Concord New South Wales Australia; ^43^ Molecular Medicine Center, Department of Medical Chemistry and Biochemistry Medical University‐Sofia Sofia Bulgaria; ^44^ Research Institute of Innovative Medical Science Sofia Bulgaria

## Abstract

**Objective:**

Biallelic variants in genes encoding the RNA polymerase III complex (Pol III) cause a spectrum of neurological disorders primarily affecting the central nervous system. Monoallelic variants have been reported in the *POLR3B* subunit only, associated with neurodevelopmental disorder, epilepsy, and peripheral neuropathy. We describe a novel Pol III‐related disorder caused by monoallelic variants in *POLR3A* and presenting primarily with peripheral neuropathy.

**Methods:**

We performed clinical and genetic investigation of the affected families. Biophysical characterization of mutant proteins was performed in silico. Immunoblotting, interactomics, and transcriptomics characterization were done in cellular models.

**Results:**

We identified 11 patients across 8 unrelated families harboring heterozygous missense variants in *POLR3A,* occurring de novo or segregating in an autosomal‐dominant fashion. The patients presented with an early‐onset, progressive sensorimotor peripheral polyneuropathy with intermediate to demyelinating ranges of nerve conduction slowing, occasionally accompanied with neurological or non‐neurological features. White matter abnormalities, characteristic for the biallelic Pol III‐related disorders, where not observed in the brain magnetic resonance imaging (MRI) evaluations. The neuropathy‐associated variants cluster in regions critical for Pol III activity. We observed mis‐regulation of individual Pol III targets and global downregulation of tRNA pools in patient‐derived cells. This Pol III dysfunction was not due to impaired POLR3A expression, subcellular localization, or subunit interactions.

**Interpretation:**

Our findings expand the Pol III‐related disease spectrum beyond the known biallelic phenotypes and establish *POLR3A* as a novel peripheral neuropathy‐associated gene. This work highlights a central role of Pol III dysfunction in disease and reinforces the link between tRNA metabolism and peripheral neurodegeneration. ANN NEUROL 2026;100:655–671

Eukaryotic RNA polymerase III (Pol III) is a 17‐subunit enzymatic complex responsible for the transcription of small noncoding RNAs, including 5S rRNA, nuclear‐encoded tRNAs, and several regulatory RNAs.^1^ Biallelic pathogenic variants in genes encoding subunits of Pol III have been reported to cause heterogeneous phenotypes primarily affecting the central nervous system (CNS). Recessive genetic alterations in *POLR3A*,^2^, ^3^ *POLR3B*,^4^ *POLR1C*,^5^ *POLR3K*,^6^ and *POLR3GL* ^7^ subunits lead to hypomyelinating leukodystrophy (HLD) and often include extra‐neurological features including abnormal dentition, endocrine, and ocular abnormalities. Biallelic variants in *POLR3A*,^8^ *POLR3B*,^9^ and *POLR3GL* ^10^ also cause Wiedemann‐Rautenstrauch syndrome (WRS), a neonatal progeroid disorder characterized by neurological impairment, including white matter abnormalities.^11^ Hypomorphic *POLR3A* variants have also been reported to cause spastic ataxia and spastic paraparesis when compound heterozygous with a pathogenic *POLR3A* variant.^12^

Whereas biallelic variants in Pol III subunit genes are well‐established as a cause of inherited neurological disease, pathogenic monoallelic variants have been reported in *POLR3B* only.^13^, ^14^, ^15^ These patients present with global developmental delay, epilepsy, ataxia, and progressive spasticity. Early‐onset, demyelinating, sensorimotor polyneuropathy is frequently observed as part of the syndrome and, occasionally, can be the only clinical presentation, classified as Charcot–Marie‐Tooth type 1I (CMT1I, MIM #619742). POLR3B constitutes the active site of Pol III together with POLR3A, the largest subunit in the enzymatic complex.^16^ Despite their shared involvement in forming the catalytic core, monoallelic pathogenic POLR3A variants have not previously been associated with a neurological phenotype.

Here, we report monoallelic POLR3A missense variants as a novel cause of peripheral neuropathy. We identified 11 patients across 8 unrelated families, 2 with autosomal dominant and 6 with de novo inheritance, consistently presenting with early onset, sensorimotor polyneuropathy either in isolation or with variable CNS or non‐neurological features. Notably, this cohort had no white matter abnormalities on brain magnetic resonance imaging (MRI), in contrast to patients with biallelic POLR3A‐related disorders. Structural modeling suggested that the affected residues localize to domains critical for Pol III enzymatic activity or nucleic acid interaction, whereas transcriptome analysis revealed dysregulation of Pol III target genes and a global reduction of the cellular tRNA pool, consistent with impaired Pol III activity. Our findings broaden the genetic and phenotypic spectrum of *POLR3A*‐related neurological disorders and have important implications for the diagnostic evaluation of patients with peripheral neuropathies.

## Materials and Methods

### Patient Cohort

The patient cohort was established through collaboration with the Inherited Neuropathy Consortium, diagnostic laboratories, GeneMatcher^17^ and GENESIS.^18^ Written informed consent was obtained from all participants or a legal guardian according to the Declaration of Helsinki. The study was approved by local ethics committees of the institutes involved. The participants underwent systematic and standardized physical, neurological, and electrophysiological examinations in their referring clinical centers. Motor nerve conduction velocity (NCV) of median nerve was categorized as slow (<25 m/s) or intermediate (25–45 m/s).^19^ Whenever available, neuroradiological data were obtained.

### Genetic Analyses

Genomic DNA was isolated from peripheral blood or buccal swab samples according to standard procedures. Exome sequencing (ES), genome sequencing (GS), or long‐read genome sequencing (lrGS) using Oxford Nanopore Technologies was performed using established protocols. Each referring center performed independent downstream bioinformatic analyses detailed in the Supporting Information.

### In Silico Analyses

The Protein Data Bank in Europe Knowledge Base (PDBe‐KB) was consulted for the 3‐dimensional structure of POLR3A in association with the Pol III elongation complex. PyMol Molecular Graphics System^20^ versions 2.5 and 3.1 was used for visualization of the retrieved PDB entries (PDB: 7AE3, 7FJJ, and 7D59). The Residue Interaction Network Generator tool^21^ version 4 was used to characterize intramolecular and intermolecular residue‐residue interactions in the cryo‐EM structure of human Pol III complex. Relative surface accessibility scores of individual residues were calculated on PDB:7AE3 with FreeSASA software v.2.0.324 using default parameters.^22^ Additional computational analyses are described in the Supporting Information.

### Cell Lines

Skin‐derived fibroblasts from the affected individuals A‐I.1, A‐II.1, A‐II.2, B‐II.1, E‐II.1, and G‐II.7, and 6 unrelated controls were cultured using standard protocols at 37°C, in humidified air containing 5% CO_2_, with passaging when >80% confluent. Peripheral blood mononuclear cells from individuals F‐I.1, F‐I.2, F‐II.1, H‐I.1, H‐I.2, and H‐II.1 were extracted using a Ficoll‐Paque gradient and transformed with Epstein–Barr virus (EBV). Lymphoblasts were maintained under humidified air at 37°C, 6% CO_2_, and passaged every 5 days. HEK293FT cells used for the proteomics analyses were cultured using standard protocols at 37°C in humidified air (5% CO_2_), with passages performed when 70 to 80% confluent.

### Immunoblotting

Cells were pelleted, lysed in RIPA buffer, and protein quantification was performed using the Pierce BCA protein assay kit (ThermoFisher). Total protein extracts were size‐separated by SDS‐PAGE, transferred onto a nitrocellulose membrane, and incubated with appropriate antibodies. The blots were developed using Pierce ECL Plus substrate (ThermoFisher) and imaged. The following antibodies were used: anti‐POLR3A (Abcam #ab96328, 1:1,000 dilution), anti‐GCN2 (Cell Signaling #3302, 1:1,000 dilution), anti‐GCN2‐phospho‐T899 (Abcam #ab75836, 1:1,000 dilution), anti‐eIF2a (Abcam #ab26197, 1:1,000 dilution), anti‐eIF2a‐phospho‐Ser51 (Cell Signaling #9721, 1:1,000 dilution), anti‐ATF4 (ProteinTech #10835‐1‐AP, 1:1,000 dilution), anti‐puromycin (Merck #MABE343, 1:1,000 dilution), anti‐α‐tubulin (Abcam #ab7291, 1:5,000 dilution), and anti‐FLAG M2 (Sigma #F3165‐1MG, 1:10,000 dilution).

### Immunofluorescence Assay

Fibroblasts from individual E‐II.1 were cultured on glass coverslips to approximately 90% confluency. Immunostaining using the primary anti‐POLR3A antibody (Abcam #ab9632, 1:250 dilution) was performed according to standard protocols. Cells were imaged with an Axio Imager Z2 fluorescence microscope equipped with ZEN 3.8 software (Zeiss).

### Affinity Purification Coupled Mass Spectrometry

Selected peripheral neuropathy‐associated variants were introduced into the cDNA of *POLR3A* using site‐directed mutagenesis, cloned into an expression vector with a C‐terminal FLAG epitope, and the plasmids were transfected into HEK293FT cells. Cells were harvested, washed, and snap‐frozen on dry ice. The pellets were solubilized in a digitonin‐containing buffer and protein concentration was determined with the Pierce BCA Protein Assay Kit (ThermoFisher). Samples underwent affinity purification to capture FLAG‐interacting proteins, as previously decribed,^23^ with modifications described in the Supporting Information. Eluted proteins underwent liquid chromatography‐tandem mass spectrometry (LC‐MS/MS) on a Q Exactive HF‐X mass spectrometer (ThermoFisher) coupled with an Ultimate 3000 RSLC nanoHPLC (Dionex Ultimate 3000), operating on data‐dependent acquisition mode. Data analysis are detailed in the Supporting Information.

### Quantitative Reverse Transcription Polymerase Chain Reaction

RNA was extracted using the miRNeasy kit (Qiagen), followed by DNase treatment (TURBO DNA‐free kit, Invitrogen). The cDNA was synthesized using the Superscript III reverse transcriptase (ThermoFisher) or iScript cDNA synthesis kit (Bio‐Rad) according to the manufacturer's instructions. The relative expression of target genes was determined by quantitative reverse transcription polymerase chain reaction (qRT‐PCR) with *PSMB6*, *SDHA*, and *COPS7A* housekeeping genes used for normalization. The qRT‐PCR was performed using SYBR Green PCR master mix (Applied Biosystems), SensiMix SYBR Hi‐ROX Kit (Meridian Bioscience) or GoTaq Green Master Mix (Promega). Expression quantification was performed using the qBase+ software (Biogazelle). The qRT‐PCR primers are listed in the Supporting Information.

### Transcriptomics

Small RNA enriched samples were extracted from the EBV‐transformed lymphoblasts of 3 affected (F‐I.1, F‐II.1, and H‐II.1) and 3 unaffected individuals (F‐II.1, H‐1.1, and H‐1.2) using miRNeasy kit (Qiagen). Two small RNAs (70 and 94 nucleotides) were synthesized and spiked into the sequencing libraries alongside the standard External RNA Controls Consortium (ERCC) mix. Libraries were prepared according to the Illumina Stranded Total RNA preparation protocol with ligation using the Ribo‐Zero Plus kit with less stringent clean‐up steps (1.2x) and sequenced on an Element Biosciences AVITI platform.

Quality control, end trimming, mapping to hg38, mapping quality assessment, gene count extraction, and expression normalization were performed following standard protocols. Gene counts were extracted in 3 successive analyses using (1) all mapped reads, (2) only primary alignments, and (3) only uniquely mapped reads (see Supporting Information). Differential expression analysis was done using DESeq2.^24^

## Results

### *Identification of* POLR3A‐*Associated Peripheral Neuropathy*

**FIGURE 1:**
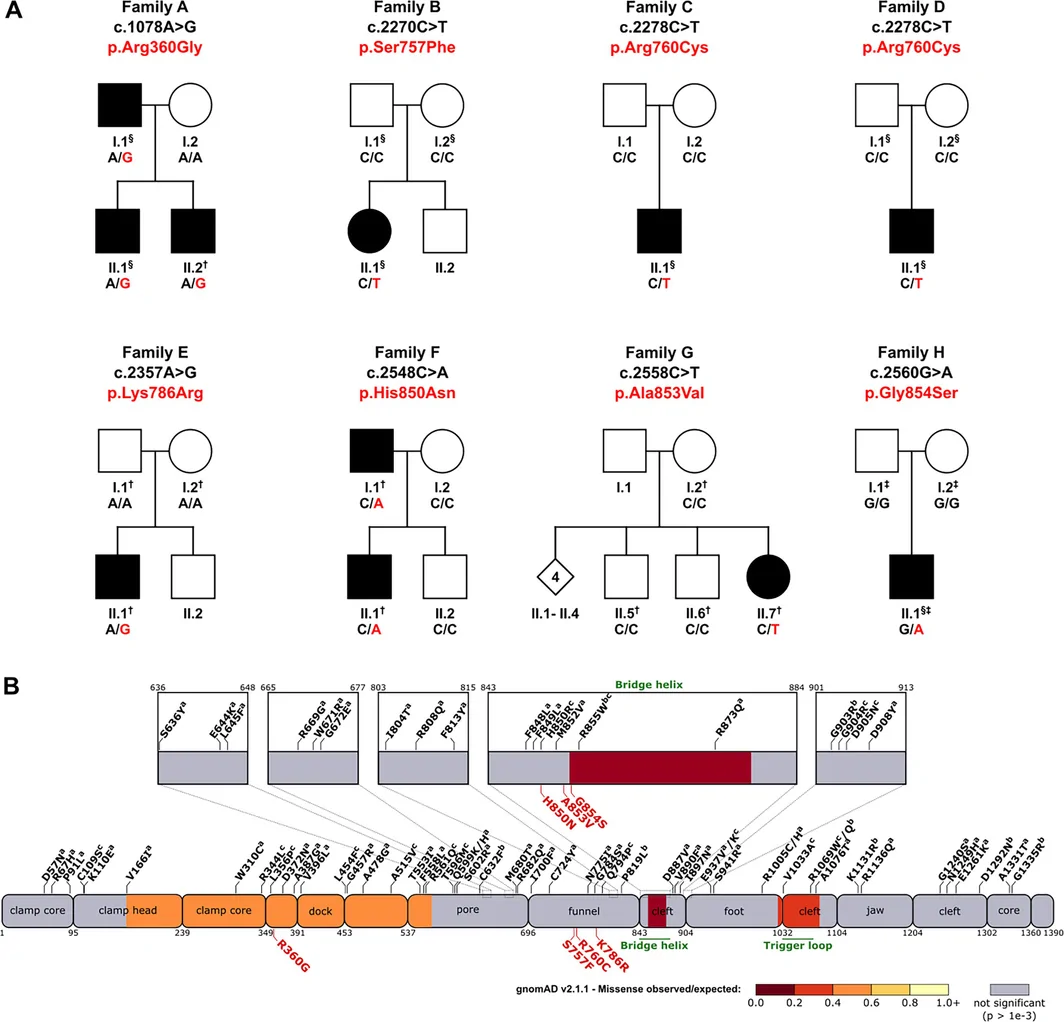
Heterozygous missense variants in POLR3A cause peripheral neuropathy. (A) Segregation analysis in pedigrees with monoallelic *POLR3A* missense variants identified in this study. Variant identification was done using ES (^§^), GS (†), or lrGS (‡) as indicated. (B) Schematic representation of the POLR3A protein sequence with missense substitutions linked to neurological phenotypes mapped onto annotated domains. Color coding reflects regional missense constraint based on data from gnomAD v.2.1.1. Substitutions in black are associated with *POLR3A*‐related biallelic disorders: (a) hypomyelinating leukodystrophy, (b) Wiedemann‐Rautenstrauch syndrome, (c) spastic ataxia/spastic paraparesis without overt hypomyelination. Substitutions identified as disease‐causing for peripheral neuropathy in this study are highlighted in red. ES = exome sequencing; GS = genome sequencing; lrGS = long‐read genome sequencing. [Color figure can be viewed at www.annalsofneurology.org]

Exome/genome sequencing of local cohorts of genetically unresolved patients with neuromuscular or neurometabolic disorders identified heterozygous variants in *POLR3A* in 4 probands by research teams in Belgium, Australia (Perth), and the Netherlands (Fig 1). Remarkably, all probands exhibited a clinical phenotype characterized by a common feature of peripheral neuropathy (Table 1 and Supplementary Table S2). This clinical overlap prompted a targeted search for additional patients with peripheral neuropathy harboring rare deleterious variants in *POLR3A*. In this way, 4 additional families were identified in Australia (Sydney), Brazil, and the United States. In all cases, the heterozygous variant was a missense substitution absent in gnomAD version 4, having a likely pathogenic prediction by AlphaMissense,^25^ and with segregation consistent with de novo or autosomal dominant inheritance. Where possible, parentage was verified in families with de novo variants (families B, D, E, and H). The variant shared by the 2 unrelated probands, C‐II.1 and D‐II.1, was shown to occur on different chromosomal backgrounds (Supplementary Fig S2). Given the well‐established association of *POLR3A* with recessive disorders, the intronic and untranslated regions of the gene were screened with no additional pathogenic variants being identified in any of the probands.

**TABLE 1:** Summary of Clinical Characteristics of the Families With Pathogenic Monoallelic *POLR3A* Variants

Family	A			B	C	D	E	F		G	H
Individual	A‐I.1	A‐II.1	A‐II.2	B‐II.1	C‐II.1	D‐II.1	E‐II.1	F‐I.1	F‐II.1	G‐II.1	H‐II.1
*POLR3A* variant	R360G	R360G	R360G	S757F	R760C	R760C	K786R	H850N	H850N	A853V	G854S
Sex	M	M	M	F	M	M	M	M	M	F	M
Age of onset	4 yr	1 yr 4 mo	13 yr	2 yr	9 mo	2 yr	8 mo	2–3 yr	1 yr	3 yr	2 yr
First sign/symptoms	GD	GD	GD	DW	DS	DW	Epi	GD	DW	DW	DW
Age at last examination	78 yr	48 yr	46 yr	32 yr	7 yr	19 yr	7 yr	60 yr	20 yr	15 yr	22 yr
Distal LL weakness	Yes	Yes	Yes	Yes	n.d.	Yes	Yes	Yes	Yes	Yes	Yes
Distal UL weakness	Yes	Yes	Yes	Yes	n.d.	Yes	No	Yes	Yes	Yes	Yes
Proximal weakness	Yes	n.d.	n.d.	Yes	n.d.	Yes	n.d.	Yes	Yes	No	Yes
Areflexia/hyporeflexia	Yes	Yes	Yes	Yes	yes	Yes	n.d.	Yes	Yes	Yes	Yes
Sensory loss	Yes	Yes	Yes	Yes	n.d.	Yes	n.d.	Yes	Yes	Yes	Yes
Loss of ambulation	No	No	No	15 yr	No	16 yr	No	53 yr	No	No	14 yr
Skeletal deformities	*pes planus*	*pes planus*	*pes planus*	Claw toes and hands	*pes planus*	Hammer toes, limitations on elbow, knee and ankle flexion	Kyphosis	*pes planus*, hammer toes	*pes planus*	*pes cavo‐varus*	Kypho‐scoliosis, *pectus carinatum*
Scapula alata	No	No	No	No	Yes	Yes	No	No	No	No	Yes
CNS involvement	No	No	No	No	Atax	ID	Epi ID	No	No	No	Atax
Brain MRI (age)^a^	n.d.	n.d.	n.d.	n.d.	N	N	n.d.	N	n.d.	n.d.	CA
Non‐neurological features	No	No	No	TA	GHD, SS	TA	TA	No	No	No	No

^a^ Age‐related abnormalities not described here.

Abbreviations: Atax = ataxia; CA = cerebellar atrophy; DS = delayed sitting; DW = delayed walking; Epi = epilepsy; F = female; GD = gait difficulty; GHD = growth hormone deficiency; ID = intellectual disability; M = male; mo = month or months; MRI = magnetic resonance imaging; N = normal; n.d. = not determined; SS = short stature; TA = teeth abnormalities; y = year or years.

All affected individuals underwent deep clinical characterization (see Table 1 and Supporting Information). The cohort included 9 male subjects and 2 female subjects, with ages at the last examination ranging between 7 and 78 years. Gross motor impairment was the initial and predominant symptom in most patients, typically presenting as delayed independent walking and/or gait difficulties, with a mean age of onset at 3.15 ± 3.4 years (range = 9 months to 13 years). Some individuals exhibited delays in earlier motor milestones, such as crawling and unsupported sitting.

**FIGURE 2:**
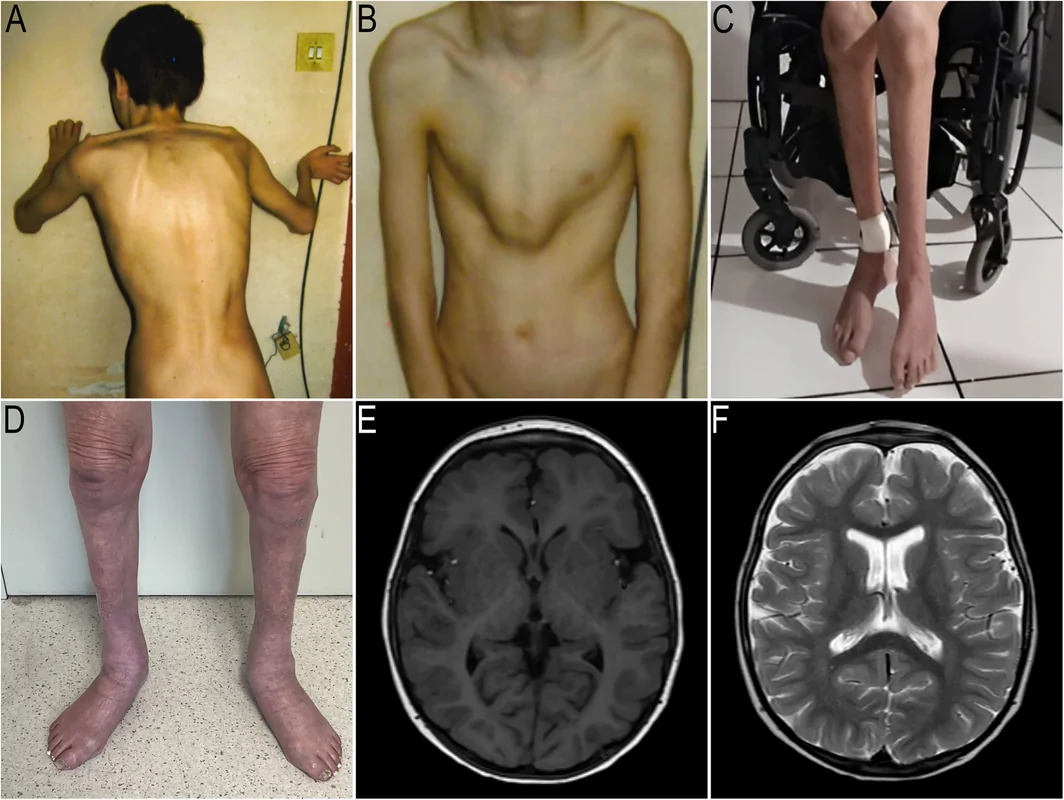
Clinical and neuroimaging features of patients with monoallelic *POLR3A* variants. (A, B) Muscle atrophy, scapula alata, kyphoscoliosis and *pectus carinatum* in patient H‐II.1 at age 12 years. (C) Muscle atrophy in lower limbs in patient D‐II.1 at age 19 years. (D) Muscle atrophy in the lower limbs in patient A‐I.1 at age 78 years. (E, F) Axial T1‐ and T2‐weighted brain MRI images of patient E‐II.1 at age 5 years showing normal white matter signal intensity for this age. MRI = magnetic resonance imaging. [Color figure can be viewed at www.annalsofneurology.org]

At their last examination, symmetrical muscle weakness was present in the lower limbs of all patients and in the upper limbs of 90% of patients for whom this data was available (see Supplementary Table S2, Appendix 1A, 1B). Deep tendon reflexes were absent in the lower limbs and/or distal upper limbs of all individuals assessed. Distal sensory loss, involving superficial and deep sensation, was uniformly reported in all tested patients. Motor and sensory deficits were usually more pronounced distally. Pyramidal signs, such as Babinski sign or spasticity, were absent in all individuals, except for E‐II.1, who had brisk tendon reflexes. Individuals D‐II.1, G‐II.7, and H‐II.1 were underweight, likely due to generalized muscle atrophy (Fig 2A–C). Scapula alata was present in 3 individuals (C‐II.1, D‐II.1, and H‐II.1). Significant skeletal deformities were reported in 45% of the patients and included claw/hammer toes, pes *cavo‐varus*, kyphoscoliosis, and *pectus carinatum* (see Fig 2B). Six patients had *pes planus*.

Although the cohort displayed consistent early disease onset, the effects on motor performance and disease progression varied significantly. Although most patients remained ambulatory, either walking independently or with assistance, 4 required a wheelchair at their latest evaluation. This variability was exemplified in patient A‐I.1, who continued to walk independently after 65 years of disease progression. In contrast, patient H‐II.1 had significant impairment of both gross and fine motor function, as well as proprioception in all extremities, and became wheelchair‐dependent at age 14 requiring assistance with transfers in his 20s (Appendix 1C). In contrast to the marked variability between families, intrafamilial differences were limited. In families A and F, which included 3 and 2 affected members, respectively, clinical manifestations were relatively uniform; for instance, both affected sons of patient A‐I.1 remained ambulatory into their fifth decade.

Nerve conduction studies (NCS) were performed at ages 6 to 78 years, revealing peripheral polyneuropathy with intermediate to demyelinating range of conduction slowing. The mean motor NCV in the median nerve was 28.6 m/s (range = 9–40 m/s) among tested patients (Table 2). Sensory nerve action potentials were undetectable in both ulnar and sural nerves in all tested patients, in accordance with the clinically observed deficits of all sensory modalities. Temporal dispersion was reported in 3 patients from 2 families (A‐I.1, A‐II.1, and H‐II.1).

**TABLE 2:** Findings From the Nerve Conduction Studies of the Families with Pathogenic Monoallelic *POLR3A* Variants

Ind.	Age at eval.	Motor: CMAP/MCV/Max onset latency				Sensory: SNAP/SCV/Max peak latency			Other findings
		Med	Uln	Per	Tib	Med	Uln	Sur	
A‐I.1	78 yr	0.4/27.9/7.1	0.5/39.9/3.9	abs	abs	abs	abs	abs	n.t.
A‐II.1	48 yr	6.9/40/4.2	3.6/41/13.3	abs	abs	abs	abs	abs	n.t.
A‐II.2	47 yr	5.4/38.9/5.5	8.3/45.1/3.2	abs	abs	abs	abs	abs	n.t.
B‐II.1	Childhood	n.a.	n.a.	n.a.	n.a.	n.a.	n.a.	n.a.	Described as demyelinating sensorimotor pattern
C‐II.1	6 yr	MCV reduced to 50%	MCV reduced to 60–70%	n.a.	n.a.	abs	abs	n.t.	Described as demyelinating sensorimotor pattern with secondary axonal degeneration
D‐II.1	24 yr	abs	abs	abs	abs	abs	abs	abs	n.t.
E‐II.1	7 yr	n.t.	n.t.	3.5/24/9.58	6.6/29.5/7.97	n.t.	n.t.	n.t.	n.t.
F‐I.1	48 yr	n.t.	abs	n.t.	n.t.	abs	abs	abs	abs lateral antebrachial cutaneous SNAP; blink reflexes 19.2 ms
F‐II.1	8 yr	4.2/31/5.1	n.t.	n.t.	n.t.	n.t.	n.t.	n.t.	n.t.
G‐II.7	10 yr	5.5/25.8/5.68	3.9/25.2/5.16	abs	abs	abs	abs	abs	n.t.
H‐II.1	9 yr	0.22/9/11	0.1/16.5/6.8	abs	abs	abs	abs	abs	n.t.

CMAP measured in millivolts (mV); MCV and SCV measured in meters per second (m/s); SNAP measured in microvolts (μV); Max peak latency measured in milliseconds (ms).

abs = absent; CMAP = compound muscle action potential; eval = evaluation; Ind = individual; med = median nerve, MCV, motor conduction velocity; n.a., not available; n.t. = not tested; per = peroneal; SNAP, Sensory nerve action potential; SCV, sensory conduction velocity; uln = ulnar nerve; tib = tibial nerve.

In addition to peripheral neuropathy, 4 patients demonstrated CNS involvement. Patients C‐II.1 and H‐II.1 displayed cerebellar ataxia, dysarthria and dysphagia. Patients D‐II.1 and E‐II.1 exhibited intellectual disability. Patient E‐II.1 presented with seizures as the initial symptom at 8 months, which subsequently progressed to myoclonic and myoclonic‐astatic epilepsy and global developmental delay.

Brain MRI data were available for 4 affected individuals, including 3 with CNS involvement (C‐II.1, E‐II.1, and H‐II.1), and one without CNS features, imaged 53 years after disease onset (F‐I.1 at age 57 years). Notably, the neuroimaging data did not show evidence of HLD or other white matter abnormalities in these individuals (Fig 2E, 2F). Cerebellar atrophy was observed in individual H‐II.1 presenting clinical signs of ataxia.

Non‐neurological features were observed in 4 patients, including short stature, growth hormone deficiency, and mild teeth abnormalities (ie, enamel defects). Interestingly, patients C‐II.1 and D‐II.1, harboring the same *POLR3A* variant, demonstrated a similar degree of motor impairment relative to their age; however, they exhibited distinct extra‐peripheral nervous system (PNS) manifestations, with the former presenting with growth hormone deficiency and the latter with intellectual disability.

### Neuropathy‐Associated POLR3A Missense Variants Impair Pol III Architecture and Function

POLR3A and POLR3B form the active center of Pol III where phospho‐diester bonds between adjacent ribonucleotides are formed using a single‐strand DNA template, whereas the newly formed DNA–RNA hybrid is translocated away from the transcription bubble to create space for the next ribonucleotides to arrive.^26^ As such, POLR3A needs to interact with nucleic acids and the surrounding Pol III subunits. We performed extensive computational and experimental studies to evaluate the impact of the identified variants on Pol III architecture and function.

**FIGURE 3:**
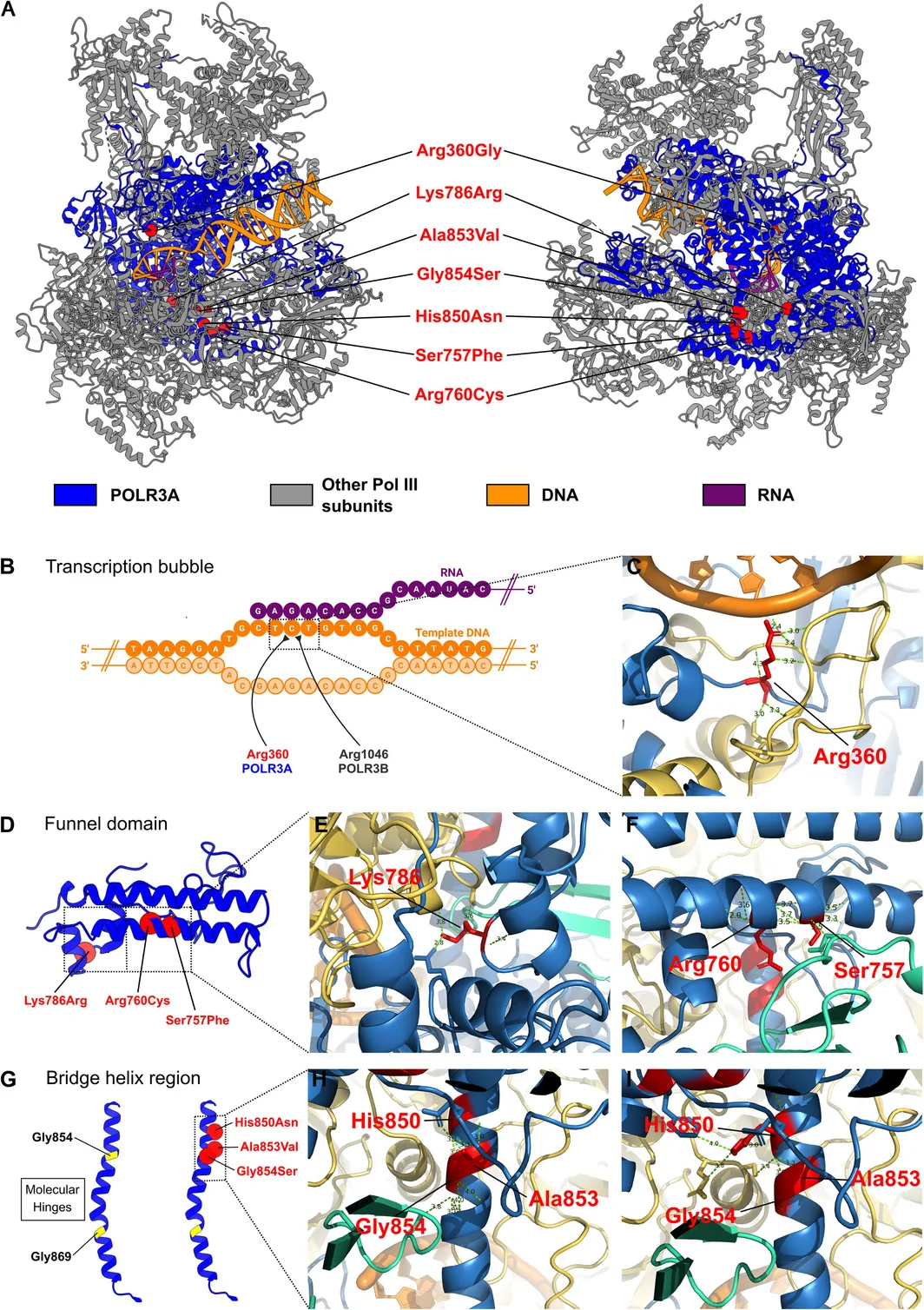
Neuropathy‐associated POLR3A residues are located in regions critical for Pol III function and make stabilizing molecular interactions. (A) The positions of the neuropathy‐associated POLR3A residues from 2 viewing angles are shown on the ribbon representation of the 3D cryo‐EM structure of the human Pol III complex (PDB: 7AE3). (B) Schematic representation of the transcription bubble highlighting neuropathy‐associated residues in POLR3A and POLR3B that contact the same nucleotide in the template DNA. (C, E, F, H, I) Intramolecular and intermolecular interactions of residues likely altered by neuropathy‐associated substitutions, indicated by dashed lines. In these panels, POLR3A, POLR3B, POLR3K, and neuropathy‐associated residues are colored blue, yellow, green, and red, respectively. (D) Ribbon representation of the POLR3A funnel domain with neuropathy‐associated POLR3A substitutions indicated in red. (G) Ribbon representation of the POLR3A BH region. Left: The BH with conserved glycine residues acting as molecular hinges (*yellow*). Right: The BH showing neuropathy‐associated POLR3A substitutions in red. BH = bridge helix.; PDB = Protein Data Bank; Pol III = polymerase III complex. [Color figure can be viewed at www.annalsofneurology.org]

Modeling the spatial location of the affected residues in the cryo‐EM structure of Pol III (PDB:7AE3) showed that they reside in the core of the complex, around the active site cleft (Fig 3A, Appendix 1D). These findings were corroborated by relative surface accessibility (RSA) calculations allowing estimation of the exposure of affected residues to solvent at the outer surface of the complex. Although all affected residues were relatively solvent‐accessible in the isolated POLR3A, their RSA scores decreased substantially when modeled within the full Pol III complex, and in the presence of nucleic acids, suggesting that these residues are buried within the assembled polymerase (Supplementary Fig S3A).

The p.His850, p.Ala853, and p.Gly854 lie within the bridge helix region and interact with each other, whereas p.Ser757 and p.Arg760 are located approximately 16 Å further from this cluster and are involved in residue‐residue interactions among themselves (see Fig 3, Supplementary Table S3). The bridge helix region is a key component of the catalytic cleft and coordinates substrate movement during transcription by kinking of 2 molecular hinges.^27^ These are conserved across archaeal, bacterial, and eukaryotic RNA polymerases,^27^ and are invariably made of glycine residues (Supplementary Fig S1A). Strikingly, p.Gly854 represents one of these hinges and is substituted with the bulkier serine residue in individual H‐II.1 (see Fig 1A). The p.Lys786 located within the funnel domain, a narrowing channel involved in transcription termination,^28^ is predicted to make interactions within POLR3A and associated subunits. Finally, p.Arg360 is located within a region connecting the clamp core and dock domains, which contributes to stabilizing DNA–RNA hybrid formation during transcription and is required for the overall stability and processivity of the elongation complex.^26^, ^28^

**FIGURE 4:**
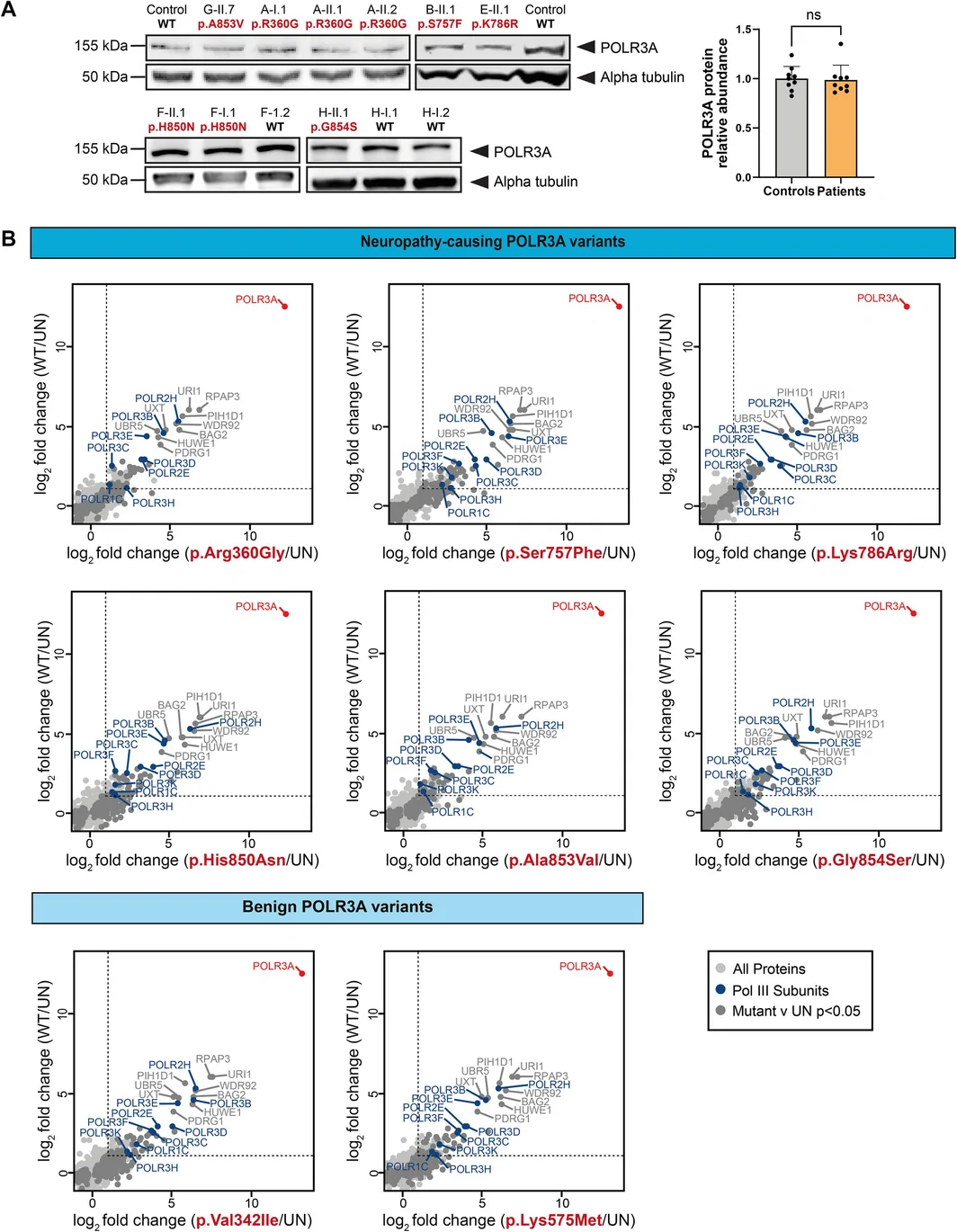
The neuropathy‐associated POLR3A substitutions do not change POLR3A abundance or Pol III complex assembly.(A): Western blot analysis of POLR3A protein levels in fibroblasts or lymphoblasts from patients in families A, B, E, F, G, and H compared with unrelated controls or unaffected family members (left). Alpha‐tubulin was used as a loading control. Quantification (*right panel*) shows no significant difference in POLR3A protein levels among 9 patients and 9 healthy control samples (unpaired *t* test; ns = not significant). (B) AP‐MS‐based interactomics profiling of FLAG‐tagged POLR3A isoforms expressed in HEK293FT cells. Correlation plots show log_2_fold‐change in protein abundance following enrichment by each neuropathy patient isoform or benign isoform of POLR3A (x‐axis) compared to wild‐type (y‐axis). Each condition was independently compared against UN controls. POLR3A is highlighted in red; other Pol III subunits are shown in dark blue. Proteins significantly enriched (mutant vs UN; *t* test *p* < 0.05) are indicated in dark grey. Dashed lines indicate a log_2_fold‐change of 1. AP‐MS = affinity purification mass spectrometry; Pol III = polymerase III complex; UN = untransfected. [Color figure can be viewed at www.annalsofneurology.org]

In addition to in silico characterization, we tested the functional impact of identified substitutions in cellular systems. Prior studies in patient‐derived fibroblasts^2^, ^3^ and HeLa^29^ models of *POLR3A*‐HLD reported that certain pathogenic biallelic variants reduce POLR3A abundance. To this end, immunoblotting analysis on neuropathy patient‐derived cells from 5 families (A, B, E, F, G, and H) established that the POLR3A abundance was comparable to that of unrelated controls or unaffected family members (Fig 4A). Furthermore, immunocytochemistry of fibroblasts derived from patient E‐II.1 revealed a similar subcellular POLR3A distribution to a control line, with predominant nuclear localization and some cytoplasmic staining (Supplementary Fig S4).

To investigate a putative impact on the POLR3A protein–protein interactions, we performed interactome profiling for each of the neuropathy‐associated alleles, except for p.Arg760Cys. For controls, we used the wild‐type protein and 2 isoforms carrying benign substitutions (p.Val342Ile and p.Lys575Met; Appendix 2A). HEK293FT cells transfected with FLAG‐tagged constructs expressing wild‐type or mutant POLR3A underwent affinity purification followed by mass spectrometry (AP‐MS). Overall, approximately 1,800 to 2,000 unique interacting proteins were detected for each condition, including 12 of 17 Pol III subunits. As expected, POLR3A was the most abundantly precipitated protein in transfected samples relative to untransfected controls (Supplementary Fig S5A, Appendix 3). Overall, neuropathy‐associated POLR3A mutants showed strong interactions with other Pol III subunits, however, these were not significantly different than interactions made by wild‐type or benign POLR3A isoforms, suggesting preserved complex assembly in the investigated variants (Fig 4B).

**FIGURE 5:**
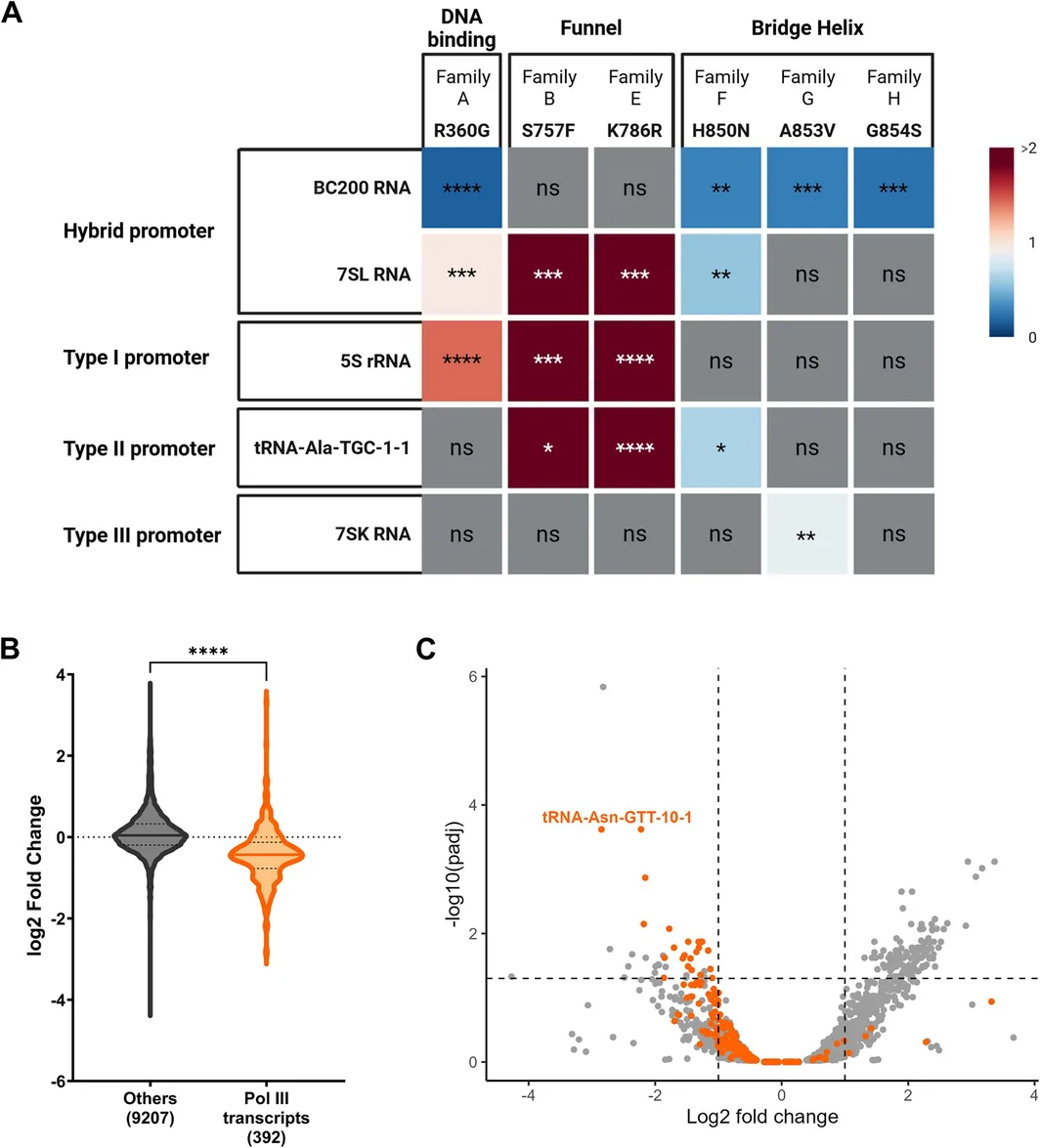
Neuropathy‐associated *POLR3A* variants impair Pol III transcription activity. (A: Graphical summary of qRT‐PCR expression analyses for selected Pol III targets across the neuropathy cohort. Significantly downregulated targets relative to controls are shown in shades of blue; significantly upregulated targets are shown in shades of red. Darker colors indicate higher fold‐change. Statistical analysis was performed using unpaired *t* tests with Welch's correction. (B) Violin plot comparing the distribution of log_2_‐fold change of transcripts synthesized by other major polymerases (n = 9,207) and transcripts synthesized by Pol III (n = 392) in 3 patients (F‐I.1, F‐II.1, and H‐II.1) relative to 3 unaffected family members (F‐I.2, H‐I.1, and H‐I.2). Pol III transcripts show a significant overall downregulation compared with other major RNA polymerases (Mean_Others_ = 0.1005, Mean_Pol III transcripts_ = −0.4436, standard error of the mean = 0.02823, unpaired *t* test with Welch's correction). (C) Volcano plot indicating differential expression of transcripts captured in the small RNA sequencing dataset comparing pooled expression in affected individuals (F‐I.1, F‐II.1, and H‐II.1) relative to unaffected family members (F‐I.2, H‐I.1, and H‐I.2). Each dot represents a distinct RNA transcript, with tRNAs highlighted in orange. Vertical dashed lines indicate a log_2_‐fold change threshold of ±1 for significantly up‐ and downregulated transcripts. The horizontal dashed line marks an adjusted *p* value cutoff of 0.05. One significantly downregulated transcript (tRNA‐Asn‐GTT‐10‐1) that is labelled on the plot was orthogonally validated in families F and H and investigated for differential expression in families A, B, E, and G, as well. *:*p* < 0.05, **:*p* < 0.01, ***:*p* < 0.001, ****:*p* < 0.0001, ns: non‐significant; Pol III = polymerase III complex; qRT‐PCR = quantitative reverse transcription polymerase chain reaction. [Color figure can be viewed at www.annalsofneurology.org]

Although the interactomics studies suggested the overall architecture of Pol III remained intact, we questioned whether its transcriptional function was impaired due to neuropathy‐associated substitutions. Using patient‐derived fibroblasts or lymphoblasts, we performed qRT‐PCR analysis of 4 Pol III targets (5S rRNA, 7SL RNA, 7SK RNA, and tRNA‐Ala‐TGC), each governed by a different promoter type^29^ and reported to be dysregulated in patients with *POLR3A*‐HLD or *POLR3A*‐WRS. BC200 RNA, a primate‐specific transcript predominantly expressed in brain tissue, was also quantified due to its putative role in the hypomyelination in HLD cellular models.^30^ The qRT‐PCR analyses revealed differential dysregulation of the Pol III targets across families depending on the substitution location. Patients with variants within the funnel domain displayed upregulation of 7SL RNA, 5S rRNA, and tRNA‐Ala‐TGC. In contrast, they showed no change in the BC200 RNA levels, whereas this transcript was consistently downregulated in all remaining patients. The patients with the variant affecting the DNA binding residue showed a hybrid profile, characterized by elevated 7SL RNA and 5S rRNA and reduced BC200 RNA expression (see Fig 5A).

We then conducted an unbiased small RNA transcriptome analysis in lymphoblasts from 3 affected (F‐I.1, F‐II.1, and H‐II.1) and 3 unaffected individuals (F‐I.2, H‐I.1, and H‐I.2) in 2 families. An average of 89.1 M reads per sample was obtained, with 87.7% meeting the Q30 threshold. The dataset included 9,599 transcripts with >20 reads in at least 3 individuals. Among these, 392 were transcribed by Pol III, including 373 precursor tRNAs representing 60% of all high‐confidence and predicted tRNA entries listed in GtRNAdb (n = 619).^31^ Compared with transcripts from other major RNA polymerases, Pol III transcripts showed a significant decrease in patients relative to unaffected family members, indicating a global downregulation for the Pol III transcriptome (Fig 5B). This decrease was mainly due to tRNA depletion, with 23 tRNAs from multiple isoacceptor families reaching statistical significance (adjusted *p* value < 0.05; Fig 5C, Appendix 2B). One significantly downregulated transcript (tRNA‐Asn‐GTT‐10‐1) was orthogonally validated in families F and H, and additionally investigated for differential expression in families A, B, E, and G using qRT‐PCR. Similar to the findings in the targeted analysis, there was upregulation of tRNA‐Asn‐GTT‐10‐1 in patients with funnel domain substitutions, whereas the individuals with bridge helix substitutions showed significant downregulation. In contrast, the patients with the DNA‐binding residue substitution showed no significant change in expression (Supplementary Fig S6). Altogether, these findings reinforce the variant‐specific trend of Pol III transcript dysregulation.

As tRNA deficiency can trigger the integrated stress response (ISR) pathway and ISR activation has been observed in the brain tissue of a *POLR3A*‐HLD mouse model,^32^ we investigated ISR markers in lymphoblasts from families F and H. Immunoblotting showed no upregulation of phospho‐GCN2, phospho‐eIF2a, or ATF4 in the patients relative to unaffected family members. Similarly, puromycin treatment did not reveal decreased global translation (Supplementary Fig S7).

## Discussion

This study identifies heterozygous missense variants in the *POLR3A* gene as a novel cause of sensorimotor polyneuropathy. Through clinical, genetic, computational, and functional analyses, we show that variants co‐segregate with disease in dominant or de novo patterns and result in a phenotype distinct from classical *POLR3A*‐related biallelic disorders.

Overall, the patients exhibited a clinically and electrophysiologically homogeneous core phenotype, characterized by an early onset, progressive sensorimotor polyneuropathy with delayed motor milestones, severe distal limb impairment, and intermediate to demyelinating range conduction slowing along peripheral nerves. The disease progression was generally severe and all patients experienced significantly impaired mobility, with approximately 40% requiring a wheelchair within 2 decades of disease onset. There was inter‐familial variability in the motor impairment and the rate of disease progression, leading to various degrees of muscle atrophy, motor disability, and skeletal deformities. A subset of patients exhibited additional CNS and extra‐neurological involvement. Although the number of affected individuals carrying variants in different regions of *POLR3A* was insufficient to establish genotype–phenotype correlations, patients with de novo variants (independent of their position in the protein) appeared to have more pronounced motor impairment and were more likely to present with extra‐PNS involvement.

Prominent clinical CNS findings were observed in fou4r patients, including cerebellar ataxia, tremor, and intellectual disability. Notably, patient E‐II.1 exhibited myoclonic‐atonic seizures from 8 months of age followed by global neurodevelopmental delay. Peripheral neuropathy was not initially reported, however, electrophysiological examination at age 7 years detected a demyelinating sensorimotor neuropathy. Epilepsy‐related gene panel testing did not identify any recurrent pathogenic variants explaining the seizures. Further cases are needed to determine if epileptic encephalopathy reflects a broader manifestation of *POLR3A*‐related monoallelic disease or a coincidental comorbidity.

Mild systemic features were observed in 4 patients, including teeth abnormalities, growth hormone deficiency, and short stature, which could be missed during routine neurological evaluations (see Table 1 and Supplementary Table S2). When present alongside early‐onset peripheral neuropathy, these non‐neurological signs may serve as clinical clues for *POLR3A*‐related neuropathy.

The clinical features observed in our cohort resemble those reported in monoallelic *POLR3B*‐related disease.^13^, ^33^ Currently, 23 individuals with heterozygous *POLR3B* missense variants have been described, typically presenting with peripheral neuropathy as a diagnostic hallmark. Extra‐PNS features (ataxia, developmental delay, and endocrine abnormalities) have been variably observed in both cohorts (Supplementary Table S4). As in patient E‐II.1, myoclonic epilepsy has occasionally appeared as the primary symptom, with subclinical peripheral neuropathy detected only through electrophysiology.^15^ These similarities support a shared clinical spectrum associated with monoallelic variants in both catalytic subunits of Pol III, centered around peripheral neuropathy.

Overall, there are notable differences in the clinical and radiological features of the monoallelic and biallelic Pol III‐related disorders. Although biallelic variants are associated with HLD or focal myelin abnormalities in milder presentations, white matter abnormalities were absent in all 4 imaged peripheral neuropathy patients in our cohort. Similarly, none of our patients showed WRS hallmarks, such as lipodystrophy or craniofacial dysmorphisms (see Supplementary Table S4). Conversely, patients with WRS and their heterozygous parents do not show PNS involvement (L. S. M. Barbosa and R. R. Arantes, personal communication). Nevertheless, some patients in our cohort exhibited additional signs overlapping with recessive Pol III‐related disorders, including cerebellar ataxia, growth hormone deficiency, and tooth abnormalities. Interestingly, other proteins involved in cytosolic tRNA biogenesis have also been implicated in both HLD and peripheral neuropathy, the prominent example being the aminoacyl‐tRNA synthetases. Biallelic variants in *EPRS1* ^34^ and *RARS1* ^35^ are associated with HLD. Contrastingly, monoallelic variants in 8 aminoacyl‐tRNA synthetases cause peripheral neuropathy.^36^, ^37^, ^38^, ^39^, ^40^, ^41^, ^42^, ^43^ In *AARS1*, monoallelic variants cause CMT,^38^ whereas biallelic variants cause an infantile‐onset encephalopathy with reduced central myelination and peripheral neuropathy^44^ – features seen across both monoallelic and biallelic Pol III‐related disorders. Taken together, additional cases and systematic clinical comparison of monoallelic and biallelic *POLR3A* cases will be essential to determine whether these conditions fall along a shared phenotypic continuum or represent distinct clinical entities.

The majority of neuropathy‐associated monoallelic variants cluster within the funnel domain and the bridge helix region of POLR3A.^28^ Presently, 9 missense variants in the funnel domain and 6 in the bridge helix are reported in biallelic *POLR3A*‐related disorders (see Fig 1B and Appendix 2A), but the specific substitutions differ between disease phenotypes. For instance, monoallelic p.His850Asn causes peripheral neuropathy, whereas p.His850Arg in compound heterozygosity with c.1909 + 22G>A causes ataxia, dental and white matter abnormalities.^45^ A qualitative comparison of amino acid changes suggests that neuropathy‐associated substitutions are more structurally disruptive than those linked to *POLR3A*‐HLD/WRS (see Supplementary Fig S3C, S3D) and cause a clinical phenotype on their own. In contrast, the substitutions associated with biallelic Pol III‐related disorders exert a clinical phenotype only in combination with a loss‐of‐function or hypomorphic allele. Additionally, certain residues are intrinsically vulnerable, such as p.Gly854, representing a hinge point in the bridge helix and associated with the monoallelic phenotype. Thus, our observations suggest that monoallelic and biallelic *POLR3A*‐related disorders involve non‐overlapping amino acid changes, and the phenotypic outcome depends on the specific substitution surpassing a critical threshold of functional impairment.

We systematically characterized monoallelic *POLR3A* variants to assess their impact on Pol III function. All affected residues are in regions essential for transcriptional activity. The p.Arg360 contacts a DNA nucleotide in the transcription bubble and is involved in DNA–RNA hybrid stabilization.^26^, ^28^ Intriguingly, this parallels the role of p.Arg1046 in POLR3B, a residue recurrently substituted in patients with monoallelic *POLR3B*‐related peripheral neuropathy.^13^, ^46^ Additionally, a cluster of neuropathy‐associated substitutions in the funnel domain interact with each other and contribute to inter‐subunit stabilization (see Fig 3). In *Schizosaccharomyces pombe*, p.Lys780Arg, orthologous to human p.Lys786Arg, increases transcription termination likely due to enhanced interaction between the substituted arginine and a glutamate residue within POLR3K.^47^ Three neuropathy‐associated substitutions are located in the bridge helix, a structural element coordinating the conformational changes in the transcription bubble through kinking of 2 molecular hinges.^27^ Systematic mutagenesis of the bridge helix residues in *Methanocaldococcus jannaschii* RNA polymerase, including orthologous residues identified in our cohort (p.His806Asn and p.Gly810Ser), result in reduced polymerase activity,^27^ supporting their deleterious impact.

Direct evidence for the damaging effect of the identified variants was obtained by evaluating the Pol III transcriptome in neuropathy patient‐derived cells. We systematically quantified the endogenous expression of 5 Pol III target transcripts, each corresponding to a distinct promotor type recognized by the polymerase complex and observed that transcript expression correlated with the topology of the affected residues. Cells carrying variants in the funnel domain showed upregulation of 7SL RNA, 5S rRNA, and tRNA‐Ala‐TGC, but not of BC200 RNA and 7SK RNA. In contrast, cells carrying variants outside this region had downregulation of BC200 RNA. This primate‐specific neural transcript is downregulated in oligodendroglia models and fibroblasts from *POLR3A*‐HLD patients carrying p.Met852Val in combination with a null allele.^28^ Interestingly, the substitution resides in the bridge helix region adjacent to the 3 neuropathy‐associated variants. Choquet et al^30^ established a causal relationship between downregulation of BC200 RNA and oligodendroglial homeostasis, as targeted knockout of this gene results in their delayed differentiation and maturation.

Additionally, we performed an unbiased small RNA sequencing in lymphoblasts from families F and H, carrying neighboring variants in the bridge helix region, and observed global decrease of the Pol III transcriptome in the affected individuals. The downregulation was pronounced for the tRNA pool, further demonstrating the Pol III dysfunction in patient cells. Notably, studies analyzing transcriptome profiles of patients with biallelic Pol III‐related disorders,^6^, ^30^, ^48^, ^49^ *POLR3A*‐HLD HeLa models,^30^ and *POLR3A*‐HLD mouse^32^ also report global reduction in (primarily premature) tRNA levels. Unfortunately, there is limited consistency between the individual tRNA genes downregulated across the studies, including ours. Notably, ISR activation due to decreased tRNA supply restricted to the peripheral neurons of mouse models of *Gars1‐* and *Yars1‐*neuropathy has been reported.^50^ We did not observe ISR activation in lymphoblasts of 3 patients with *POLR3A‐*neuropathy, however, a neuron‐specific effect cannot be excluded. Overall, our findings indicating downregulated tRNA pools lend support to the emerging concept of disrupted tRNA metabolism as a driver of neurodegeneration in both the PNS and CNS.

Population data from gnomAD v4 show that *POLR3A* exhibits low constraint against protein‐truncating variants (pLi = 0; LOEUF = 0.836), and no neurological phenotypes have been reported in heterozygous carriers of truncating variants. This, combined with our findings that POLR3A abundance and subcellular localization are not altered in neuropathy patient‐derived cells (see Fig 4A and Supplementary Fig S4), excludes haploinsufficiency as a mechanism underlying *POLR3A‐*neuropathy. Additionally, our interactome findings suggest that defective subunit interactions leading to incomplete Pol III complex assembly is not the overarching disease mechanism for *POLR3A‐*related peripheral neuropathy. The identified monoallelic variants may act through an alternative dominant‐negative mechanism (eg, disrupted interactions with DNA template) or may exert a gain‐of‐function effect, the precise nature of which remains unknown. A limitation of the interactome studies is that only monoallelic *POLR3A* variants were assessed for effects on Pol III complex assembly. Future studies examining a broader range of variants, including biallelic *POLR3A* variants, will enable more robust comparisons of subunit assembly across different Pol III‐subunit variants and associated phenotypes, and may help clarify mechanistic differences underlying monoallelic versus biallelic Pol III‐related disease.

In conclusion, we demonstrate that monoallelic missense variants in *POLR3A* cause early‐onset peripheral neuropathy, with or without extra‐PNS involvement. These findings expand the clinical spectrum of *POLR3A*‐related disorders beyond the well‐characterized biallelic phenotypes and warrant careful consideration or reinterpretation of heterozygous variants in this gene in patients with peripheral neuropathy. Our study provides critical insights into the allelic diversity of *POLR3A*‐related disorders and establishes a framework for understanding the molecular pathomechanisms underlying these conditions. Further mechanistic studies are needed to understand why these monoallelic variants primarily affect the PNS, which may reveal fundamental links between RNA metabolism and human disease.

## Author Contributions

M.L.K., M.M.O., G.R., and A.J. contributed to the conception and design of the study; L.L.P.R., J.M.P., R.W., B.R.G., T.L., L.M., S.V., K.R.K., D.Y., L.I.R., L.D.B., A.D.V., D.A.K., T.G., A.C., K.I., F.K., F.B.F., A.A.D.S.C., L.S.S.M.B., R.R.A., T.R., J.E.B., E.P.W., J.E.S., G.M., M.K., I.C., M.E., G.P.S., E.M., R.V.B., C.B.M., I.T., S.Z., S.J.W., C.D.M.V.K., N.L., L.N.S., D.A.S., D.N.H., V.G., and Ay.Ca. contributed to the acquisition and analysis of data; L.L.P.R., J.M.P., R.W., B.R.G., T.L., M.L.K., M.M.O., G.R., Ay.Ca., and A.J. contributed to drafting the text or preparing the figures.

## Potential Conflicts of Interest

F.K. is the co‐founder and F.B.F. is an employee of Mendelics Genomics Analysis, a private genetic diagnostics company where 2 families with monoallelic *POLR3A* variants were genetically diagnosed. The remaining authors report no competing interests.

## Supporting information


**Appendix 1A** Patient C‐II.1 at age 13 years demonstrating an ataxic gait. Proximal lower‐limb weakness is evident when rising from a crouched position.

**Appendix 1B** Patient C‐II.1 at age 13 years demonstrating distal upper‐limb weakness during eating.

**Appendix 1C** Patient H‐II.1 at age 12 years walking with assistance and demonstrating an ataxic gait.

**Appendix 1D** Video showing a 360 degrees rotation around the PDB:7AE3 structure of Pol III with neuropathy‐associated residues marked in red.

**Appendix 2A:** List of missense variants in POLR3A associated with a phenotype and a summary of in silico findings per each variant.
**Appendix 2B:** Differentially expressed genes identified through the DESeq2 analysis in the small RNAseq dataset of families F and H for primary alignments. Genes with adjusted *p* values < 0.05 are shown.

**Appendix 3A:** Unfiltered and unimputed log₂ LFQ intensity values for all proteins detected by affinity purification mass spectrometry (AP‐MS) across individual replicates. These values were used as input *t* test analysis.
**Appendix 3B:** Two‐sided *t* test dataset for each wild‐type or mutant FLAG‐POLR3A cell line against untransfected HEK293FT cells, used in Figure 4B and Supplementary Figure S4A and S4C. Proteins detected in at least 2 FLAG‐POLR3A replicates were used for analysis, and missing values from untransfected replicates were imputed based on normal distribution.
**Appendix 3C:** Two‐sided *t* test dataset for each mutant FLAG‐POLR3A cell line against Wildtype FLAG‐POLR3A cells, used in Supplementary Figure S4B. Log2 LFQ intensities were normalized to POLR3A level prior to statistical analysis. Proteins detected in 2 mutant replicates were used for analysis, and missing values from wild‐type replicates were imputed based on normal distribution.

**Data S1.** Supporting Information.

## Data Availability

Data generated during this study are included in the manuscript and its Supporting Information. Further information can be requested from the corresponding authors.
